# Fabricating Inner Channels in Laser Additive Manufacturing Process via Thin-Plate-Preplacing Method

**DOI:** 10.3390/ma16196406

**Published:** 2023-09-26

**Authors:** Junke Jiao, Shengyuan Sun, Zifa Xu, Jiale Wang, Liyuan Sheng, Jicheng Gao

**Affiliations:** 1 School of Mechanical Engineering, Yangzhou University, Yangzhou 225009, China; mz120210866@stu.yzu.edu.cn (S.S.); mz120220915@stu.yzu.edu.cn (J.W.); 006382@yzu.edu.cn (J.G.); 2 Laser Institute, Qilu University of Technology (Shandong Academy of Sciences), Jinan 250353, China; xuzifatrubo@163.com; 3 PKU-HKUST ShenZhen-HongKong Institution, Shenzhen 518057, China

**Keywords:** hybrid manufacturing, surface quality, microstructure, roughness

## Abstract

This paper presents a hybrid manufacturing process for the preparation of complex cavity structure parts with high surface quality. Firstly, laser precision packaging technology is utilized to accurately connect a thin plate to a substrate with microchannel. Secondly, Direct Metal Laser-Sintering (DMLS) technology is utilized to completely shape the part. The morphology and microstructure of laser encapsulated specimens and DMLS molded parts were investigated. The results show that the thin plate and the substrate can form a good metallurgical bond. The lowest surface roughness of the DMLS molded parts was 1.18 μm. The perpendicularity between the top of the microchannel and the side wall was optimal when the laser power was 240 W. Consequently, the hybrid manufacturing process effectively solves the problems of poor surface quality and powder sticking of closed inner cavities. The method effectively eliminates the defects of adhesive powder in the inner cavity of the DMLS microchannel, improves the finish, and solves the problem that mechanical tools cannot be processed inside the microchannel, which lays the foundation for the research of DMLS high-quality microchannel process.

## 1. Introduction

Laser Powder Bed Fusion of Metals (PBF-LB/M) technology has become one of the most promising advanced technologies due to its advantages of near-net shaping [1,2,3]. Taking a high-energy laser beam as the energy source, it realizes the rapid and accurate molding of three-dimensional parts through layer-by-layer superposition [4]. It is gradually applied to all kinds of parts preparation. Therefore, researchers are increasingly focusing on the preparation process of various complex shapes and high quality parts. However, the dimensional accuracy, geometric precision, and surface quality of DMLS parts are not as good as conventionally machined parts, which has hindered the widespread use of this new method [5,6,7]. One of the key issues with this technology is the deleterious surface quality of the produced parts, which usually requires post-processing. To solve this problem, post-processing such as milling, sandblasting, and polishing are usually performed [8,9,10].

Many scholars have proposed additive and subtractive hybrid manufacturing technology as a solution to address this problem. Gong et al. [11] utilized additive/subtractive hybrid manufacturing technology to investigate the densification level, microstructure, micro-hardness, and residual stress characterization in different zones of the part by manufacturing 316L SS specimens. Liou et al. [12] proposed a multi-axis laser cladding hybrid processing method that combines a five-axis laser cladding system with a mechanical milling machining center to achieve additive manufacturing of arbitrarily complex shapes by rotating the table, which improves the processing efficiency. Liu et al. [13] developed a hybrid selective laser melting (SLM)/CNC milling system and validated it using stainless steel 316L, resulting in nearly full dense parts with improved dimensional accuracy and surface roughness. Li et al. [14] proposed a six-axis robotic arm with additive manufacturing and subtractive-machining heads to achieve the additive and subtractive hybrid manufacturing process. Soshi et al. [15] demonstrated a rapid fabrication of an injection mold with conformal cooling channels using laser deposition and mechanical machines, improving the cooling performance. Yan et al. [16] successfully produced TC4 thin-wall parts with good build strategy through the hybrid manufacturing process. In the study by Liu et al. [17,18], hybrid additive manufacturing was proposed to improve the surface quality of additive manufacturing components, and it was found that laser polishing greatly reduced the surface roughness and improved the mechanical properties.

Yang et al. [19] successfully prepared high-quality 316L parts using a combination of laser directed energy deposition additive and hot milling cut-off technologies. Through the optimization of process parameters, the microstructure of the parts was refined, and the density was high, showing high micro-hardness (246.73 HV) and tensile strength (683.3 MPa). Du et al. [20] emphasized that additive/subtractive composite manufacturing technology is an effective method for producing high-performance and complex aerospace parts. They specifically applied this technology to the production of martensitic aging steel parts with high density, surface quality, shape, and size accuracy. Jeng et al. [21] developed a new process by combining the selective laser cladding process with the traditional milling process for mold manufacturing and repair. Pan et al. [22] proposed the plasma deposition and milling composite manufacturing process, which greatly enhanced processing efficiency and accuracy by removing plasma deposition layers using conventional milling in a layer-by-layer manner. Additionally, Tian et al. [23] successfully designed and implemented a five-axis additive/subtractive composite processing equipment, enabling the additive/subtractive composite manufacturing of three-dimensional metal parts, including high-temperature alloys, high-entropy alloys, and titanium alloys.

The above research mainly follows the approach of combining laser manufacturing with traditional mechanical processing. However, there have been few studies conducted on the hybrid laser manufacturing of complex inner surface parts, particularly for closed cavities with intricate and precise shapes. Laser processing can effectively address issues such as severe tool wear and poor resolution. The stability of the machining process and the overall quality control of complex internal cavity parts, however, are difficult to achieve.

Therefore, we have investigated the impact of DMLS on thin-walled packages and its effects on the surface quality and microstructure of the top region within the inner cavity. In this paper, a hybrid process of laser precision packaging and DMLS is used to prepare microchannel. Firstly, the cross-sectional morphology of the sheet combined with the substrate after laser packaging was analyzed. Secondly, the effects of four sets of DMLS laser power on the morphology and microstructure of the microchannel inner wall were investigated.

## 2. Materials and Methods

### 2.1. Materials

**Figure 1:**
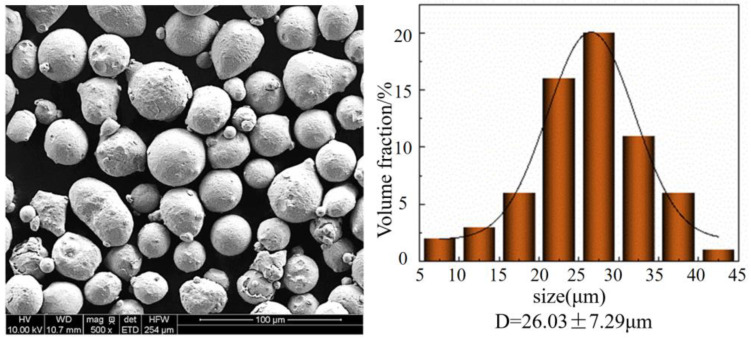
SEM and powder distribution of 316L SS powders.

316L SS powders (Chengdu Huayin Powder Technology Co., Ltd., Chengdu, China) were manufactured through gas-atomization and used in the DMLS manufacturing parts. The chemical composition of the powders is provided in Table 1. Figure 1 demonstrates the surface morphology and particle size distribution of the 316L SS powders, with the majority of the powders exhibiting spherical shapes. Figure 1 indicates that 80% of the fine powder had a diameter range of 18 μm to 33 μm, with an average particle size of D determined as 26.03 μm. Dry the powder to 80 °C and keep warm for two hours before the experiment to ensure that the powder does not contain moisture, to avoid the influence of DMLS. The substrate is a 316L plate with micro-grooves of 1 mm depth and width on the substrate. A step of 0.1 mm in depth and 0.05 mm in width was reserved on both sides of the microgroove. The pre-placed thin plates were 316L with width and thickness of 1.1 mm and 0.1 mm, respectively.

### 2.2. Hybrid Manufacturing Process

**Figure 2:**
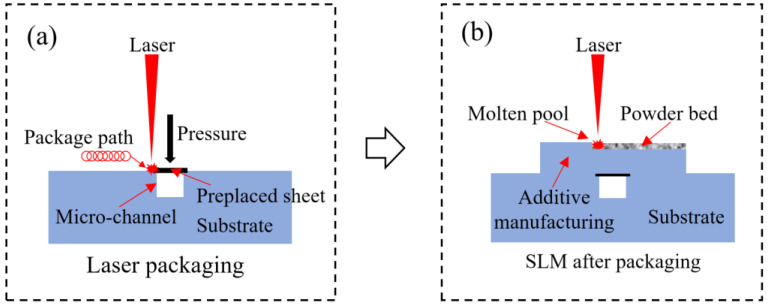
The schematic diagram of hybrid manufacturing process: (**a**) laser precision packaging; (**b**) DMLS after packaging.

The hybrid manufacturing experiments were carried out using a continuous wave (CW) laser precision packaging system (Maxphotonics, Shenzhen, China) and DMLS system (EOS, Munich, Germany), as shown in Figure 2. The continuous wave laser precision packaging system consists of a continuous wave laser of power P = 1000 W and wavelength λ = 1060 nm, a scanning galvanometer, a motion control system, and an inert gas protection system. DMLS system using EOS-M290 (Beijing Hengshang Technology Co., Ltd., Beijing, China) (Yb-fiber fiber laser peak power P_pk_ = 400 W, spot diameter D = 10–500 μm, maximum scanning speed V_max_ = 7 m/s, layer thickness range 20–100 μm, maximum molding size 250 mm × 250 mm × 325 mm). The experiment was divided into two stages: laser precision packaging and DMLS manufacturing. First, the thin plate was placed on the matrix channel and pressurized to carry out laser precision packaging test. The test parameters are laser power of P = 500 W, a scanning speed of v = 100 mm/s, a defocusing amount of Δf = 10 mm, rotation radius of δ = 1 mm, and a laser diameter of d = 0.15 mm. Then, DMLS manufacturing was performed on the packaged and formed samples, and four groups of samples were prepared by changing the laser power in the test, and the corresponding parameters are shown in Table 2, where P is the laser power, V is the laser scanning speed, Δ is the laser scanning distance, and d is the laser spot diameter. Finally, the wire electrical discharge machining technology is used for cutting and sample preparation. The morphology and microstructure were observed via scanning electron microscope (SEM) FEI Quanta FEG 250(FEI, San Jose, CA, USA) and laser scanning confocal microscope (LSCM) Keyence VK-X200 K (Keyence, Tokyo, Japan).

## 3. Results and Discussion

### 3.1. Surface Topography and Microstructure of the Laser Precision Packaging Specimens

**Figure 3:**
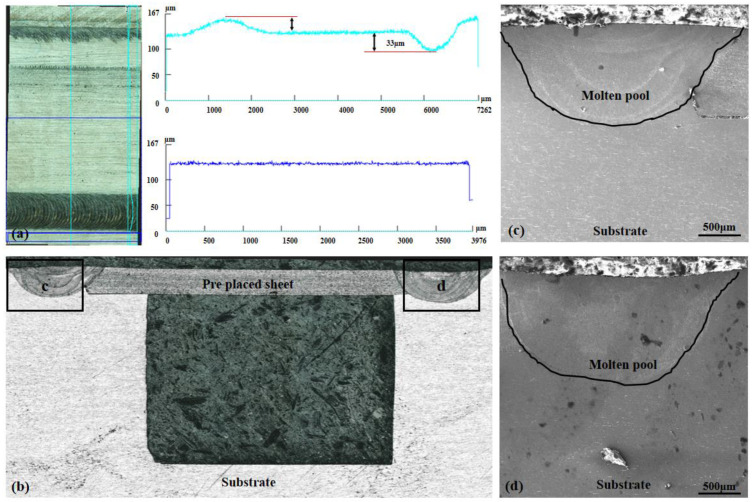
The surface topography and microstructure of the laser precision packaging specimens: (**a**) Thin plate surface morphology; (**b**–**d**) Packaging area micro-morphology.

Surface topography and microstructure of the laser precision packaging specimens were examined in this study (Figure 3). We used laser precision packaging to seal the top of the micro-channel on the substrate and to precisely connect the two sides of the micro-channel with a thin plate.

The analysis results showed that the 0.1mm thin-walled part on the surface of the micro-channel was precisely packaged with better surface quality compared to other areas. However, we observed some irregularities in the form of bumps and depressions in the weld bead, which were attributed to deformation caused by thermal stress in the packaging area. Specifically, we measured a 33 μm deep dent on the right side of the package zone and a 25 μm high bump in the left packaging area experimentally. To assess the microstructure of the packaging area, we utilized scanning electron microscopy (SEM) (Figure 3c,d). The microstructure analysis revealed no notable defects between the prefabricated thin plate and the substrate, with a metallurgical bonding observed between them.

The morphology of the packaging surface, as shown in Figure 3a,b, is relatively flat. The prefabricated thin plate exhibits minimal deformation, with only slight molten pool depressions and protrusions observed in the weld area. This suggests that effective control of thermal stress was achieved during the welding process. Since the DMLS technology can melt the powder together with the substrate and then cool and mold it, the effect of these faint bumpy areas on the subsequent DMLS process is negligible. An analysis of the weld seam (Figure 3c,d) reveals that a metallurgical bond was formed between the prefabricated thin plate and the substrate. Furthermore, almost no pores or crack defects were found in the weld seam, indicating a successful welding outcome.

### 3.2. Surface Topography and Microstructure of the DMLS

**Figure 4:**
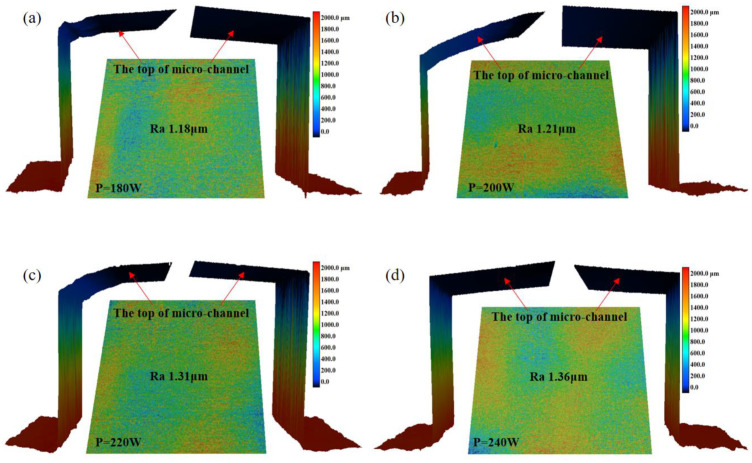
Influence of different laser powers on the top morphology of microchannels in 316L parts: (**a**) 180 W; (**b**) 200 W; (**c**) 220 W; (**d**) 240 W.

**Figure 5:**
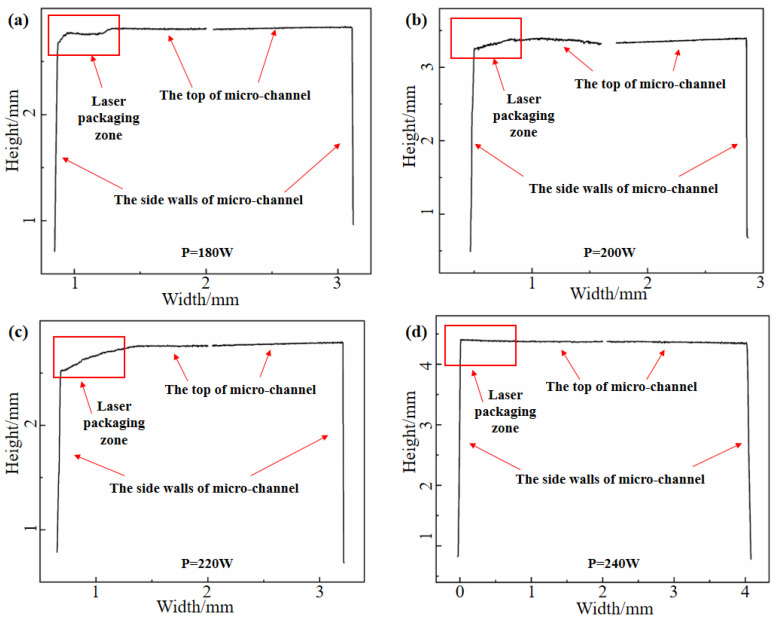
Influence of different laser powers on the microchannel profile of 316L parts: (**a**) 180 W; (**b**) 200 W; (**c**) 220 W; (**d**) 240 W.

The influence of laser power on the top morphology of micro-channels in thin-walled packages produced through DMLS processes was examined after laser precision packaging. Laser powers of 180 W, 200 W, 220 W, and 240 W were employed, as shown in Table 2, to conduct DMLS manufacturing on pre-placed thin plates. Figure 4 presents the outcome of these experiments, illustrating the effect of laser power on the top morphology of micro-channels of 316L SS parts manufactured via hybrid laser precision packaging and DMLS. The experimental findings revealed that the roughness of the top layer of the micro-channel increased as the laser power increased. Specifically, the roughness values (Ra) were 1.18 μm, 1.21 μm, 1.31 μm, and 1.36 μm for power outputs of 180 W, 200 W, 220 W, and 240 W, respectively. The roughness grade reaches approximately Class 7 accuracy, the state in which machining marks can be seen on the surface of machined parts, but not felt by hand. Low surface roughness means a high surface finish, which helps reduce stress concentrations, increase part fatigue strength, and extend service life. However, the change in roughness was not significant, suggesting that the impact of laser power on roughness is relatively small. By observing the top and sidewall profiles of the inner channel shown in Figure 5, it is clear that the top morphology varies under different powers. This variation is a result of the different thermal effects caused by the diverse heating effects of laser inputs at different power levels on the preset thin plate. Notably, at a power of 240 W, the top of the inner channel appears to be the straightest. The performance of the microchannel was affected by its accuracy, and the size accuracy of the microchannel was influenced by the DLMS parameters greatly. To this end, reducing the thermal effect on the microchannel in DMLS process becomes very important. As mentioned above, the best accuracy of the channel can be obtained when the laser power is 240 W, the laser scanning speed is 920 mm/s, the laser scanning distance is 0.12 mm, and the laser spot diameter is 30 μm.

**Figure 6:**
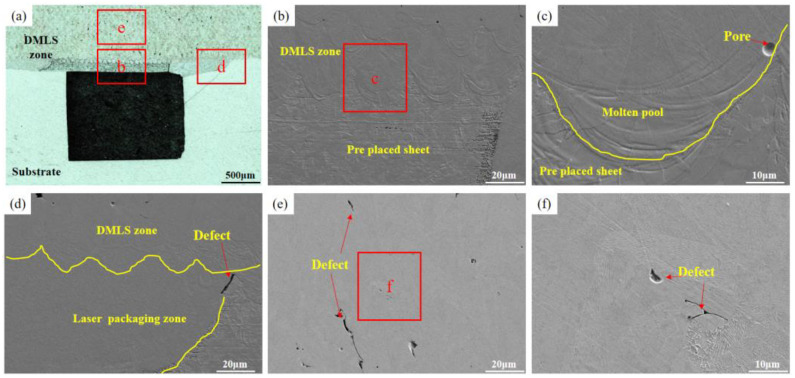
The microstructures when the DMLS laser power is 180 W: (**a**) DMLS areas macro-morphology; (**b**,**c**) Microscopic morphology of the upper layer of the packaging area; (**d**–**f**) DMLS areas with microscopic defects.

**Figure 7:**
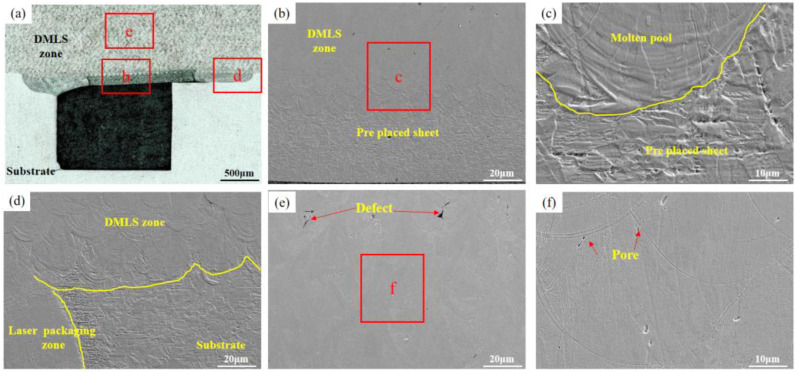
The microstructures when the DMLS laser power is 200 W: (**a**) DMLS areas macro-morphology; (**b**,**c**) Microscopic morphology of the upper layer of the packaging area; (**d**–**f**) DMLS areas with microscopic defects.

**Figure 8:**
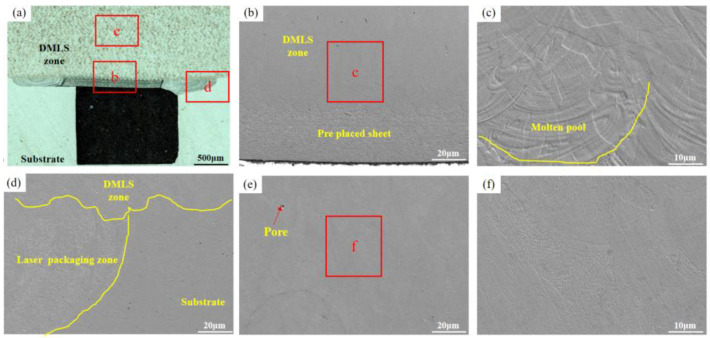
The microstructures when the DMLS laser power is 220 W: (**a**) DMLS areas macro-morphology; (**b**,**c**) Microscopic morphology of the upper layer of the packaging area; (**d**–**f**) DMLS areas with microscopic defects.

**Figure 9:**
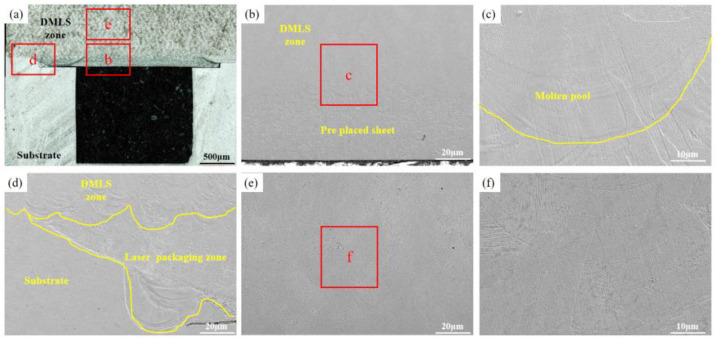
The microstructures when the DMLS laser power is 240 W: (**a**) DMLS areas macro-morphology; (**b**,**c**) Microscopic morphology of the upper layer of the packaging area; (**d**–**f**) DMLS areas with microscopic defects.

In order to systematically explore the effect of laser power on the overall quality of DMLS manufacturing specimens, LSCM and SEM were used to observe the specimen microstructure. The cross-sectional morphology of the specimens under different laser powers is shown in Figure 6, Figure 7, Figure 8 and Figure 9. We observed that a good metallurgical bond could be formed between the DMLS zone and the thin plate (Figure 6b, Figure 7b, Figure 8b and Figure 9b). The metallurgical bond allows the molten powder particles to diffuse into the matrix, thus increasing the bond strength between the both. Further magnification of the bonding zone between the DMLS zone and the thin plate reveals that the quality of the boundary of the molten pool after cooling and molding is poor at the power of 180 W and 200 W (Figure 6c and Figure 7c), and pores are present at the boundary of the molten pool at 180 W. Smaller unfused defects are present at the junction of the DMLS zone, the laser packaging zone, and the substrate at 180 W (Figure 6d), which may be caused by poorly bonding the thin plate to the substrate during the laser precision packaging process. It may be caused by a poor fit of the thin plate to the substrate during the laser precision packaging process.

In addition, the presence of a large number of pores and defects in the DMLS manufacturing zone (Figure 6e) seriously affects the mechanical properties of the parts, which is due to the low heat input. With the increase in laser power, the number of pores and defects in the DMLS zone decreases gradually. When the laser power is 200 W and 220 W, the number of pores and defects in the DMLS layer decreases and becomes smaller, as shown in Figure 7e and Figure 8e. As the laser power was further increased to 240 W, no obvious pores and defects were seen in the sample cross-section (Figure 9e), and its density was the highest among the four samples. The above defects were mainly attributed to the low laser power, which resulted in insufficient heat input and failure of the powder to melt sufficiently, ultimately producing porosity and defects. It is worth noting that the size, number, morphology, and location of the pores have an important effect on the mechanical properties of the components, a higher porosity will shorten the fatigue life of the molded parts, and the pores close to the surface have a greater effect on the fatigue properties of the molded parts than any other location. Therefore, the best quality of DMLS molding was achieved with the laser power of 240 W, scanning speed of 920 mm/s, hatching distance of 0.12 mm, and spot diameter of 30 μm. These pores and defects are mainly caused by the gas in the molten pool escaping too late and the unstable shape of the molten pool. Subsequently, process parameters (scanning speed, spot size, defocusing, etc.) can be optimized to ensure the sealing of the inert gas protected space.

**Figure 10:**
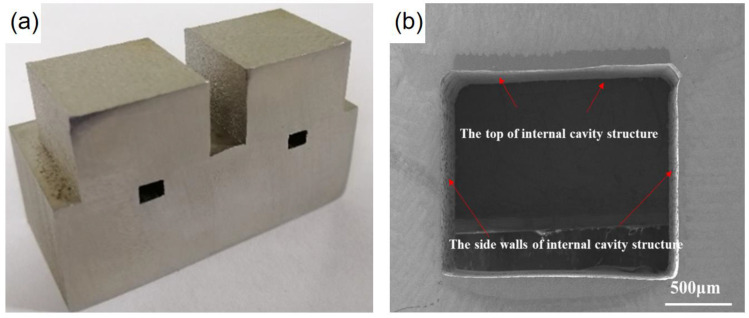
(**a**) Microchannel structural parts fabricated via laser hybrid manufacturing; (**b**) Cross-sectional morphology of microchannel.

As mentioned above, the DMLS parameters not only influence the accuracy of the microchannel, but also affect the microstructure and the thermal defects of the component, which affect the performance and the mechanical properties of the component. And the best parameter is the laser power of 240 W, scanning speed of 920 mm/s, hatching distance of 0.12 mm, and spot diameter of 30 μm. With this optimized parameter, a microchannel was produced as below. After connecting the thin plate to the two sides of the microchannel via laser precision encapsulation, the microchannel structural member was successfully formed with high quality by using DMLS technology (EOS, Munich, Germany) for manufacturing on the thin plate (Figure 10a). The top surface of the internal cavity does not produce defects such as unmelted particles, the sidewalls are smooth, and the high-quality connection between the top and the side parts can also be realized as shown in Figure 10b. Moreover, the optimization of process parameters minimizes the deformation generated in the packaging area, and the DMLS manufacturing area has no obvious defects and forms a good metallurgical bond with the thin plate and substrate area. The microchannel prepared via this hybrid method has potential applications in the field of microchannels for aero-engine radiators, air-conditioning chillers, water-cooling circuits for large-scale equipment, etc.

## 4. Conclusions

In this paper, a novel hybrid manufacturing process is proposed to prepare 316L microchannel structural parts with high surface quality. The quality of the parts after laser precision package molding was observed. Then, DMLS manufacturing was carried out on this basis to explore the effects of different process parameters on the top morphology and microstructure of the inner cavity. The main experimental results and analysis of this study can be summarized as follows:

(1) The laser precision packaging process can effectively form a metallurgical bond between the thin plate and the substrate, which improves the bonding strength between the thin plate and the substrate. Under the effect of thermal stress, the left and right sides of the packaging area show a convex mark of 25 μm and a pit of 33 μm, respectively.
(2) By optimizing the DMLS process parameters (P = 180 W, V = 920 mm/s, d = 30 μm, Δ = 0.12mm), the surface roughness of the complex cavity was greatly reduced to Ra 1.18 μm.
(3) When the laser power is 240 W, the top of the internal channel seems to be most perpendicular to the sidewalls. And the DMLS areas have the least defects such as porosity.
(4) The hybrid manufacturing process successfully solved the problems of poor surface quality and powder adhesion in the closed inner cavity, which provides a reference for the research process of the manufactured microchannel.

## Figures and Tables

**Table 1:** Chemical composition of 316L SS powders (mass fraction %).

Element	Cr	Ni	Mo	Mn	Si	C	Fe
316L SS powder	17.09	10.61	2.38	1.17	0.59	0.013	Bal

**Table 2:** Parameters for DMLS.

No.	P/W	V/mm/s	Δ/mm	d/μm
1	180	920	0.12	30
2	200	920	0.12	30
3	220	920	0.12	30
4	240	920	0.12	30

## Data Availability

Data sharing is not applicable.
